# Antituberculosis Macozinone Extended-Release Tablets To Enhance Bioavailability: a Pilot Pharmacokinetic Study in Beagle Dogs

**DOI:** 10.1128/spectrum.02327-22

**Published:** 2022-12-12

**Authors:** Angela Koryakova, Victoria Shcherbakova, Olga Riabova, Yurii Kazaishvili, Roman Bolgarin, Vadim Makarov

**Affiliations:** a Chemical Diversity Research Institute, Khimki, Russia; b Nearmedic Pharma LLC, Obninsk, Russia; c Federal Research Centre “Fundamentals of Biotechnology” of the Russian Academy of Sciences (Research Centre of Biotechnology RAS), Moscow, Russia; Rutgers University

**Keywords:** macozinone, PBTZ169, antituberculosis drug, extended-release tablet, pharmacokinetics, oral bioavailability

## Abstract

Macozinone (MCZ; PBTZ169) is a first-in-class antituberculosis clinical-stage benzothiazinone-based drug candidate. Although its efficacy and safety have been strongly proven in several preclinical and clinical studies, the physicochemical and pharmacokinetic properties specific to MCZ required further optimization. Accordingly, this study aimed to evaluate the pharmacokinetics of MCZ administered as extended-release (ER) tablets F2 and F6 compared to immediate-release (IR) dispersible tablets for oral suspension. Oral absorption of MCZ from ER tablets was significantly different from that of IR tablets after a single oral dose in Beagle dogs in both fasted and fed states. In addition, food directly affects the bioavailability of MCZ from ER tablets but does not affect it from IR tablets. The high values of relative bioavailability of the prolonged-release tablets F2 and F6 compared to the IR tablets may indicate an indirect confirmation of their gastroretentive properties. Taken together, pharmacokinetic parameters have demonstrated that these MCZ oral formulations not just enhance drug bioavailability but may also improve regimen adherence by reducing MCZ dose frequency and reducing the development of drug resistance.

**IMPORTANCE** Macozinone (MCZ) is the newest first-in-class clinical-stage benzothiazinone-based drug candidate for the treatment of tuberculosis. Yet, the extremely low oral bioavailability of MCZ, a major problem in clinical trials, needed to be addressed, and we are pleased to present our attempts to solve this issue. We report that extended-release tablets of MCZ significantly increased key pharmacokinetic parameters in the preclinical setting. We suggest that these MCZ oral formulations not just enhance drug bioavailability but may also improve regimen adherence by reducing MCZ dose frequency and reducing the development of drug resistance.

## INTRODUCTION

**FIG 1:**
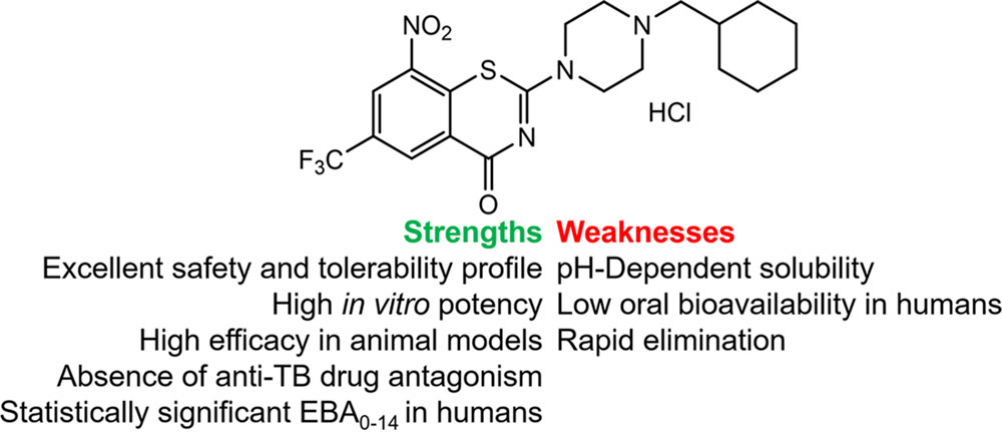
Chemical structure of macozinone hydrochloride and its features requiring optimization.

Macozinone (MCZ; formerly PBTZ169·HCl; Fig. 1) is an advanced antituberculosis drug candidate belonging to the chemical class of benzothiazinones (1). The molecule inhibits the synthesis of mycobacterial cell wall by covalent targeting the essential enzyme decaprenylphosphoryl-β-d-ribose-2′-epimerase (DprE1) at nanomolar concentrations (MICs ~0.2 ng/mL) (2–7). The antibacterial potency of benzothiazinone-based molecule derivatives is directly proportional to their lipophilicity (4), but the high lipophilicity of a drug contributes to its poor water solubility and variable oral bioavailability (8). Moreover, the water solubility of macozinone strongly depends on the pH level: it highly dissolves in strongly acidic solutions (pH ~1 to 2) and poorly dissolves in moderately acidic solutions (pH ~5 to 6) (9). In biorelevant media, macozinone is more soluble in fed-state simulated intestinal fluid (FeSSIF) than in fasted-state simulated intestinal fluid (FaSSIF). Further, MCZ shows low permeability in the Caco-2 assay (an early drug discovery assay that uses a human colon epithelial cancer cell line as a model for human intestinal absorption of an experimental compound) with an efflux ratio of 0.6 (9). The pharmacokinetics (PK) of macozinone immediate-release dispersible tablet for oral suspension in Beagle dogs after a single oral administration of 160 mg supported the primary *in vitro* findings on the narrow absorption window of the drug, the maximum drug concentration in plasma (*C*_max_) and the total drug exposure (AUC_inf_) in the fed state are 1.8 and 1.3 times, respectively, higher than in the fasted states: absorption is confined to the upper gastrointestinal tract (9). In addition, macozinone is rapidly eliminated, the mean residence time (MRT_last_) was low (3.6 h) and the half-life (*t*_1/2_) was short (3.2 h) (9). Guo and colleagues (10) reported low absolute bioavailability of macozinone (*F*_abs_ ~10%) in mice after a single oral administration of 12.5 or 25.0 mg/kg. Despite this, macozinone demonstrates low toxicity and remarkable efficacy in various animal models of Mycobacterium tuberculosis infection (2), so the molecule was promoted into the first-in-human studies.

The favorable safety and tolerability profiles MCZ have been confirmed in several randomized, double-blind, placebo-controlled clinical trials in which the drug was administered as immediate-release oral dosage forms, a native crystal powder for solution (clinicaltrials.gov, Phase Ia, NCT03423030 and Phase Ib, NCT03776500) or a capsule (clinicaltrials.gov, Phase Ia, NCT03036163 and Phase Ib, NCT04150224). However, the pharmacokinetics of macozinone immediate-release capsules from Phase I confirmed fast absorption and rapid elimination of the drug, indicating the need for frequent dosing to maintain antimycobacterial efficacy (clinicaltrials.gov, NCT03036163 and NCT04150224). A preliminary food intake is important to increase the effectiveness of MCZ: the basic absorption, distribution, metabolism, elimination (ADME) parameters statistically significantly improved when the drug is taken in the fed state. High-dose administration of macozinone beyond 640 mg did not lead to a further increase in oral bioavailability, which averaged 12% (clinicaltrials.gov, NCT03036163 and NCT04150224).

All these unsuitable physicochemical and pharmacokinetic features specific to macozinone require further optimization (Fig. 1). According to the Biopharmaceutical Classification System, macozinone is considered a class IV drug owing to its low water solubility and permeability (9). Hence, MCZ has been proposed as a suitable candidate for a mucoadhesive gastroretentive (or extended-release [ER]) drug delivery system. Nesterenko et al. (11) developed 10 batches of ER tablets and selected two experimental formulations (microcrystalline cellulose [F2] or 2-hydroxypropyl-β-cyclodextrin [F6]) based on their favorable drug release level, swelling, and mucoadhesive properties *in vitro*.

In this pilot study, we compared the pharmacokinetics (PK) of MCZ administered as previously developed immediate-release dispersible tablets and two types of extended-release (F2 and F6) tablets in healthy Beagle dogs after a single oral dose. We also evaluated their PK in both fed and fasted states to select the best dosage form for macozinone. In addition, we determined the relative bioavailability of the new MCZ oral formulations.

## RESULTS AND DISCUSSION

Discovered in 2014, macozinone represents a safe and effective antituberculosis drug candidate with a low rate of resistance development. An earlier dose-ranging study Phase IIa of the early bactericidal activity (EBA) of PBTZ169 was conducted over 14 days in a small group of sputum smear-positive pulmonary-tuberculosis patients using MCZ doses of 160 mg, 320 mg, and 640 mg per day in capsules (clinicaltrials.gov, NCT03334734). The data showed MCZ was well tolerated over the dose range evaluated, and the resulting EBA_0–14_ was statistically significant after macozinone monotherapy at 640 mg/day with a mean daily fall in CFU of Mycobacterium tuberculosis of 0.071 log_10_ CFU/day/mL sputum.

Yet, the extremely low oral bioavailability of macozinone represents a major challenge for clinical use. Such an unsatisfactory level is a consequence of the pH-dependent solubility of the drug in the gastrointestinal tract. The drug is highly soluble at pH 1 to 4 (lower stomach), modestly soluble at pH 5 to 6 (upper stomach), and insoluble at pH 7 and above (intestines). The formation of various water-soluble salts of macozinone, the use of surfactants (sodium lauryl sulfate, Tween 80, or Triton X-100), or particle size reduction has been applied and failed to increase the solubility and bioavailability of this drug candidate (our unpublished data). Increasing the daily dose of MCZ also does not reach its suitable plasma levels. Further, in an attempt to improve its key pharmacokinetic parameters, we developed an immediate-release dispersible tablet for oral suspension, but macozinone bioavailability has scarcely improved, and the problem remained unresolved (9). Moreover, this MCZ formulation has been associated with high variability of plasma drug concentrations that potentially may lead to increased adverse events and reduced patient compliance.

Based on the results of macozinone pharmacokinetics in humans, we have determined that a suitable oral drug dosage form for this drug candidate should have a longer release at a specific site of the upper gastrointestinal tract, where the pH level promotes the high absorption and solubility of macozinone (9). Earlier, we hypothesized that an oral mucoadhesive gastroretentive tablet, i.e., a tablet capable of adhesion to the surface of mucous tissues and retention in the gastrointestinal tract, would help to overcome the present macozinone-associated challenges (11). These oral dosage forms are designed for gastric retention and modified release of an active substance (12). By interacting with the mucous membrane layer, these systems enhance the residence time of the drug and, therefore, help to increase its bioavailability. Mucoadhesive gastroretentive formulations are often used for drugs with a narrow absorption window in the upper gastrointestinal tract, low solubility/instability at alkaline pH, or poor absorption from the lower gastrointestinal tract (13, 14) - macozinone matches.

Tablets were designed using microcrystalline cellulose (F2) or 2-hydroxypropyl-β-cyclodextrin (F6) to modulate drug release (11) (Table S1 in the supplemental material). The experimental formulation F2 was swellable and mucoadhesive, and macozinone was released moderately in simulated gastric fluid, about 26% in 6 h. The other formulation, F6, greatly enhanced macozinone release *in vitro* (about 58% in 6 h) and was considered an optimized dosage form due to good mucoadhesive work (6.7 J/m^2^), mucoadhesive time (>300 min), and swelling index (99.3%) (11) (Table S1). Thus, these tablets have shown promising *in vitro* results that provide a basis for further *in vivo* pharmacokinetic studies.

From a pharmacokinetic standpoint, the rational consideration for the selection of an appropriate animal model is based on the similarity to human pharmacokinetics. There are generally two animal species are used, one rodent (mice, rats, hamsters) and one nonrodent (dogs, pigs, nonhuman primates), in preclinical absorption, distribution, metabolism, excretion, toxicology (ADME-T) studies according to the regulatory guidance (15). Whereas the use of a rodent model to assess the impact of a drug formulation on bioavailability is very limited, a nonrodent model is considered the most suitable animal model to predict the bioavailability of an experimental drug dosage form (16, 17). For our pilot study, we chose the Beagle dog model as being relatively inexpensive and commercially available.

### Extended-release tablet effect on the pharmacokinetics of macozinone.

**FIG 2:**
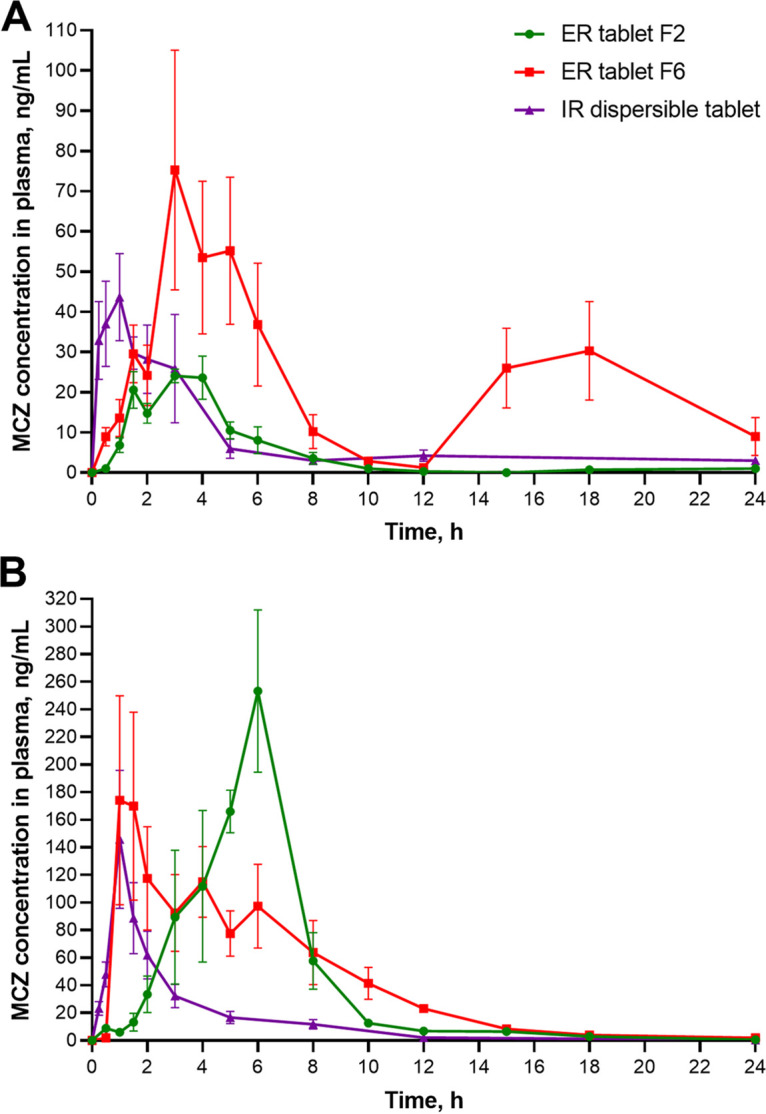
Pharmacokinetic curves for extended-release tablets F2/F6 (500 mg) and immediate-release dispersible tablet (160 mg) in fasted (A) and fed (B) states after a single oral dose in Beagle dogs (*n *= 5). Plasma concentrations are expressed as mean ± standard error of the mean (SEM).

To investigate whether macozinone mucoadhesive gastroretentive tablets could actually improve macozinone bioavailability, we compared the pharmacokinetics of two tablets, F2 and F6, after a single oral 500-mg dose in both fed and fasted Beagle dogs. All macozinone formulations tested were well tolerated by animals; no adverse effects were observed in any experimental groups. Mean plasma concentration-time curves are shown in Fig. 2, and individual plasma concentration curves are shown in Fig. S2.

The absorption kinetics for both macozinone extended-release tablets F2 and F6 in the fasted state are very different (Fig. 2). MCZ from the formulation F2 was poorly absorbed, and plasma concentration decreased monophasic with a mean maximum concentration (*C*_max_) of 31.6 ± 5.9 ng/mL (Fig. 2A). In contrast, the pharmacokinetic curve for the ER tablet F6 shows biphasic pattern: the mean peak plasma concentration of macozinone 92.1 ± 49.9 ng/mL was achieved at the mean to maximum concentration (*T*_max,1st peak_) value of 3.0 h, then the drug concentration steadily declined up to 12 h, and a secondary broad peak was observed 14 to 22 h later (*T*_max,2nd peak_ ~18 h) after drug administration (Fig. 2A). Only the plasma concentration of MCZ in one animal receiving the extended-release tablet F6 did not show the same pattern. We suggested that this secondary peak was directly related to the prescribed feeding of the animals (see “Pharmacokinetic study design” below). The mean proportion of the area under the plasma concentration-time curve from time 0 to 12 h (AUC_0–12_) value (as a percentage of plasma concentration-time curve from time zero to infinity [AUC_0-∞_]) was 59.5%, and the proportion of the area under the plasma concentration-time curve from 12 h to 24 h (AUC_12–24_) value was 36.0% (Table S2). For the ER tablet F2, the mean proportion of the AUC_0–12_ value was 95.4% (Table S2) indicating that macozinone was mainly absorbed up to 12 h. The mean residence time (MRT_last_) for the ER tablet F6 was much longer than that of the ER tablet F2 or the IR dispersible tablet (Table 1). Compared to the IR dispersible tablet, the ER tablets F2 and F6 were absorbed (median *T*_max_ 3.0 h for ERs versus 1.0 h for IR) and released the drug more slowly (median time span that plasma concentration is equal to or greater than half the value of the maximum concentration [*T*_50%_*C*_max_] 3.2/3.7 h for the ER tablets versus 1.7 h for the IR tablet) (Table 1).

**TABLE 1:** Pharmacokinetic parameters of macozinone extended-release tablets F2 and F6 and immediate-release dispersible tablet after single oral administration in Beagle dogs^*a*^

PK parameter	MCZ oral formulation and state of food intake^*b*^					
	ER F2, fasted	ER F6, fasted	ER F2, fed	ER F6, fed	IR, fasted^*c*^	IR, fed
AUC_0–∞_ (h·ng/mL)						
Mean ± SD	108.0 ± 40.0	550.4 ± 322.7^*d*^	982.4 ± 60.8^*e*^	1,021.4 ± 68.4^*e*^	254.8 ± 105.3	398.0 ± 146.4
Median	105.0	593.0	963.0	1,055.0	273.5	435.0
Range	62.0–156.0	71.8–934.0	924.0–1,076.0	928.0–1,080.0	114.0–358.0	193.0–528.0
AUC_last_ (h·ng/mL)						
Mean ± SD	106.0 ± 40.0	528.1 ± 316.7^*d*^	976.2 ± 62.8^*e*^	1,008.4 ± 62.8^*e*^	215.4 ± 97.9	322.8 ± 197.7
Median	103.0	522.0	961.0	1,040.0	223.0	390.0
Range	59.5–154.0	70.4–927.0	908.0–1,069.0	962.0–1,063.0	100.0–350.0	50.2–520.0
*C*_max_ (ng/mL)						
Mean ± SD	31.6 ± 5.9	922 ± 50.0	302.6 ± 74.1^*e*^	253.2 ± 94.6	53.5 ± 15.2	146.1 ± 111.1
Median	30.0	181.1	313.0	204.5	53.4	142.0
Range	24.2–38.2	45.8–177.0	184.0–384.0	199.0–419.0	36.9–74.5	20.1–299.0
*T*_max_ (h)						
Mean ± SD	2.8 ± 1.3	5.8 ± 6.8	5.4 ± 0.9	2.7 ± 2.2	1.3 ± 1.1	0.9 ± 0.2
Median	3.0	3.0	6.0	1.5	1.0	1.0
Range	1.5–4.0	2.0–18.0	4.0–6.0	1.0–6.0	0.3–3.0	0.5–1.0
*k*_el_ (h^−1^)						
Mean ± SD	0.46 ± 0.07^*f*^	0.46 ± 0.09^*f*^	0.22 ± 0.08	0.16 ± 0.03	0.12 ± 0.04	0.23 ± 0.08
Median	0.42	0.49	0.18	0.16	0.11	0.23
Range	0.40–0.57	0.36–0.57	0.14–0.33	0.11–0.20	0.08–0.17	0.17–0.30
*t*_1/2_ (h)						
Mean ± SD	1.56 ± 0.25^*f*^	1.56 ± 0.36^*f*^	3.48 ± 1.19	4.54 ± 1.02	6.38 ± 2.17	3.35 ± 1.13
Median	1.70	1.40	3.96	4.34	6.15	3.25
Range	1.20–1.80	1.20–2.00	2.13–4.92	3.50–6.05	4.00–9.20	2.30–4.60
MRT_last_ (h)						
Mean ± SD	4.20 ± 1.24	9.08 ± 5.01^*d*^	5.95 ± 0.69	5.65 ± 1.61	6.28 ± 1.04	3.64 ± 0.43
Median	3.70	8.30	6.04	5.95	6.30	3.50
Range	2.90–5.80	2.20–15.90	5.03–6.54	3.46–7.84	5.10–7.90	3.20–4.30
*T*_50%_*С*_max_ (h)						
Mean ± SD	2.82 ± 1.39	3.92 ± 2.07	2.87 ± 1.02	3.00 ± 1.64	2.22 ± 1.02	1.38 ± 0.39
Median	3.20	3.70	2.46	3.80	200	1.50
Range	0.90–4.60	1.20–7.0	2.20–2.74	1.07–4.50	1.20–3.60	0.80–1.80

a Extended release tablets F2 and F6 (ER), 500 mg; immediate-release dispersible tablet (IR), 160 mg; *n = *5.

b Statistical analysis was by one-way ANOVA with Tukey’s multiple-comparison test.

c Data are from reference 9.

d *P* < 0.05, compared to the ER F2 tablet-fasted group.

e *P* < 0.05, compared to the IR tablet-fed group.

f *P* < 0.05, compared to the IR tablet-fasted group.

### Food effect on the pharmacokinetics of macozinone.

Administration of a drug along with food may greatly change the ADME properties of a drug: food may enhance the absorption for some drugs or, in contrast, may attenuate the absorption for others drugs; food may delay or accelerate the absorption for drugs; finally, food may have no effect of the absorption of a drug (18–20).

Clinical studies of macozinone revealed statistically significant increases in key ADME parameters, such as mean residence time and half-life, in fed patients (NCT04150224). The present comparative pharmacokinetic study of the two macozinone extended-release oral formulations strongly supported the direct food effect on the bioavailability of the drug candidate.

We observed that the absorption kinetics for both macozinone extended-release tablets F2 and F6 in the fed state are actually vary from those in the fasted state (Fig. 2; Fig. S3). MCZ from the experimental formulation F2 is delayed absorbed: the median maximum plasma concentration was shifted from 3.0 h to 6.0 h and the mean residence time (MRT_last_) value was increased (*P* = 0.0254) (Fig. 2; Fig. S3A). In addition, the area under the plasma concentration-time curve from time zero to time of last measurable concentration (AUC_last_) and *C*_max_ values were significantly increased −9.2 and 9.6 times (*P <* 0.0001, for both; Tables 1 and 2), respectively, compared to the fasted state. In contrast, we noted earlier absorption (1 to 2 h after administration) and gentler plasma concentration reduction without secondary peaks when animals were orally administered the prolonged-release formulation F6 (Fig. 2; Fig. S3B). The AUC_last_ and *C*_max_ values for the ER tablet F6 were 1.9 (*P* = 0.0104) and 2.7 (*P* = 0.0099) times higher, respectively, than those of the fasted state. The greater absorption enhancement of the MCZ extended-release tablets in Beagle dogs after food may be due to delayed gastric emptying and gastrointestinal transit, which promotes more complete dissolution and longer residence time in the lower stomach favoring absorption.

**TABLE 2:** Food effect on key pharmacokinetic parameters of the MCZ oral formulations studied^*a*^

MCZ oral formulation/PK parameter	State of food intake		Fed/fasted ratio
	Fasted	Fed	
ER tablet F2			
AUC_last_ (h·ng/mL)	106.1 ± 40.0	976.2 ± 62.8	9.2
*C*_max_ (ng/mL)	31.6 ± 5.9	302.6 ± 74.1	9.6
ER tablet F6			
AUC_last_ (h·ng/mL)	528.1 ± 316.7	1,008.4 ± 62.8	1.9
*C*_max_ (ng/mL)	92.2 ± 50.1	253.2 ± 94.6	2.7
IR tablet			
AUC_last_ (h·ng/mL)	196.4 ± 96.5	322.8 ± 197.7	1.6
*C*_max_ (ng/mL)	57.6 ± 19.8	146.1 ± 111.1	2.6

a Values are mean ± SD.

It is known that the gastrointestinal motility and transit are different for Beagle dogs compared to humans: the total transit in dogs is about 6 to 8 h, while the total gastrointestinal transit in humans is longer and is about 20 to 30 h (21). Moreover, the pH value of the dog stomach is higher than that of the human (21). Based on this, we suggested that the absorption of MCZ from extended-release tablets may be different in clinical trials, since the time in the gastrointestinal tract will be longer and will enable longer drug release in the gastrointestinal tract, where acidic pH promotes more complete dissolution of macozinone.

In contrast, the food-effect study involving administration of immediate-release dispersible tablet under fasting and fed conditions indicated that the AUC_last_ and *C*_max_ values were only modestly increased (*P* = 0.3079 and 0.1021, respectively, nonstatistically significant differences; Table 2); therefore, the food does not facilitate the pharmacokinetics of macozinone when it was administered as the IR tablet.

### Relative bioavailability evaluation.

Table 3 presents the mean values of oral relative bioavailability for each extended-release tablet. Relative bioavailability was calculated based on the immediate-release tablet as the reference (see “Pharmacokinetic parameters” below). In the fasted state, the relative bioavailability of extended-release tablets F2 and F6 was 17.3% and 86.0% greater, respectively. The relative bioavailability of prolonged-release tablets F2 and F6 in the fed state increased significantly to 96.8% and 100%, respectively. These findings support not only the direct food effect on the pharmacokinetics of macozinone but also the gastroretentive properties of the extended-release tablets F2 and F6.

**TABLE 3:** Mean relative bioavailability of macozinone extended-release tablets F2 and F6 compared to immediate-release dispersible tablet

MCZ oral formulation	MCZ dose, mg	Relative bioavailability (*F*_rel_) %	
		Fasted state	Fed state
ER tablet F2	500	17.3	96.8
ER tablet F6	500	86.0	100.0
IR tablet	160		

### Conclusions.

There are a few limitations to the present study. First, we observed that ultrasonography was an unacceptable method to determine *in vivo* gastric retention of the drug formulation: the tablets F2 and F6 were not visible after 2 h due to their swelling coupled with the presence of dog food, but no X-ray, magnetic resonance fluorescence analysis imaging, or gastroscopy was performed to locate the tablets. The second limitation is the small (*n *= 5) sample sizes of Beagle dog groups receiving macozinone extended-release tablets. However, our study provides a proof-of-concept for these tablets to enhance the bioavailability of macozinone.

We found that the extended-release tablet F6 greatly improved the pharmacokinetic parameters of macozinone compared to the immediate-release dispersible tablet in the fasted state. As expected, food significantly contributes to the absorption and bioavailability of macozinone administered as extended-release tablet: the AUC_last_ and *C*_max_ values were increased 9.2 and 9.6 times for tablet F2 and 1.9 and 2.7 times for tablet F6 in the fed state. Collectively, our results may serve as a benchmark for further research on this extended-release oral formulation in human clinical trials to improve antituberculosis macozinone compliance by reducing drug dosage and frequency of its administration.

## MATERIALS AND METHODS

### Ethics statement.

All animal work was conducted under protocols approved by the Animal Care and Use Committee at the Chemical Diversity Research Institute (protocol no. 15/2019 from 25/10/2019) according to the center’s guidelines for animal use and the state industry standards GOST33044-2014, GOST 33215-2014, and GOST 33216-2014 (in harmonization with the European Directive 2010/63/ЕС).

### Animals.

Twenty-five 7- to 8-month-old male Beagle dogs weighing 14.6 ± 0.9 kg were purchased from the All-Russian Scientific Center for Safety of Biologically Active Compounds (Staraya Kupavna, Moscow region, Russia). Before the experiment, the animals were kept in enclosures of 2 to 3 individuals and were fed standard dry dog food “Prokhvost” (Russia) and water *ad libitum*. The animals were not euthanized as part of the experiment.

### Macozinone tablets.

MCZ immediate-release (IR) dispersible tablets for oral suspension (320 mg) were manufactured and provided by R&D Center “NovaMedica Innotech” (Moscow, Russia). MCZ extended-release (ER) tablets (500 mg) were previously formulated by wet granulation process using microcrystalline cellulose, polysorbate 80, povidone 90, polyethylene glycol, sodium carboxymethylcellulose, carbopol 974P, hydroxyethylcellulose in a mixture of water/alcohol, and sodium stearyl fumarate as excipients (11). All quality control testing and *in vitro* characterization of ER formulations have been previously reported (11).

### Macozinone administration.

Extended-release tablets F2 and F6 (500 mg of MCZ) were administered orally, one tablet per animal, followed by a fixed volume of deionized water (7 mL) to swallow the tablet. The immediate-release dispersible tablet (320 mg of MCZ) in 100 mL of deionized water was stirred using a magnetic mixer (Ohaus, USA) until a homogeneous suspension was obtained. The MCZ suspension was administered to the animal in five doses of 10 mL (total 50 mL, or 160 mg of MCZ per animal) via an oral gavage *ex tempore*. The remaining suspension was disposed of.

### Pharmacokinetic study design.

For the pharmacokinetic study, the healthy animals were randomized into five groups (five animals in each group). The level of body weight dispersion between and within the animal groups did not exceed ±15%. One oral macozinone ER tablet F2 or F6 (500 mg) was administered to two groups of animals in either the fasted or fed condition (four groups in total). The fifth animal group received the IR tablet (160 mg), which was used as the reference drug formulation (Table 4). Two animal groups were fasted for at least 8 h without access to water for at least 1 h before administration of ER tablets F2 or F6. Another three animal groups were fed dry dog food and then immediately received ER tablets F2 or F6 or an IR tablet. In fasted animal groups, free access to water was allowed after 2 h and the dog food after 10 h postmacozinone administration. In fed animal groups, free access to water was allowed immediately postmacozinone administration. The blood samples were collected at 0, 0.25, 0.5, 1, 1.5, 2, 3, 5, 8, 12, and 24 h (IR) or at 0, 0.5, 1, 1.5, 2, 3, 4, 5, 6, 8, 10, 12, 15, 18, and 24 h (ER) and centrifuged (10,000 rpm × 10 min), and the plasma was stored at −70°C before analysis.

**TABLE 4:** Pharmacokinetic study design for comparative formulation study

MCZ formulation	MCZ dose per dog	Administration route	State of food intake	No. of dogs
Extended-release tablet F2	500 mg	Oral, single dose	Fasted	5
Extended-release tablet F6	500 mg	Oral, single dose	Fasted	5
Extended-release tablet F2	500 mg	Oral, single dose	Fed	5
Extended-release tablet F6	500 mg	Oral, single dose	Fed	5
Immediate-release tablet, dispersible tablet for oral suspension	160 mg (half of 320 mg)	Oral, single dose	Fed	5

### Plasma bioanalysis.

Macozinone concentrations in plasma were determined using a validated high-performance liquid chromatography (Agilent Infinity 1290)-tandem mass spectrometry (Sciex QTRAP 5500) (HPLC-MS/MS) method in the multiple reaction monitoring (MRM) mode. The chromatographic separation was carried out at 40°C on YMC-Triart C_18_ column (50 × 2 mm, 1.9 μm) using a mobile phase A composed of acetonitrile, water, and 0.05 M ammonium acetate supplemented with 0.05 M acetic acid (90:10 vol/vol) and a mobile phase B composed of water and 0.05 M ammonium acetate supplemented with 0.05 M acetic acid at a flow rate of 0.5 mL/min; 5.0 μL of sample was injected into the HPLC-MS/MS system. The total run time was 5 min. Deuterated macozinone PBTZ169-d11 was used as an internal standard (IS). The HPLC-MS/MS data were processed using Analyst 1.6.2 software (Sciex, USA).

All plasma samples were prepared by protein precipitation with acetonitrile. Briefly, 5 μL of a 10-fold solution of PBTZ169 in acetonitrile was added to 45 μL of plasma sample and vortexed for 5 sec. Then, 150 μL of IS (100 ng/mL) was added, vortexed for 5 sec, and stored at 4°C for 15 min for protein precipitation. After centrifugation at 3,500 rpm for 15 min, 130 μL of supernatant was injected into the HPLC system. All samples were injected and analyzed in duplicate.

The bioanalytical method used was validated according to the European Medicines Agency requirements (22) and showed the following validation parameters: the linear concentration range was 0.5 to 2,000 ng/mL, and the accuracy and precision were determined at four concentration levels and ranged from 90.1 to 92.2% and from 2.03 to 11.4%, respectively. The lower limit of quantification for macozinone was 0.5 ng/mL. Representative MRM chromatograms of plasma samples from dogs showing selectivity and sensitivity of the bioanalytical method for quantification of MCZ are presented in Fig. S1.

### Pharmacokinetic parameters.

Plasma concentration-time data were analyzed by a noncompartment model using WinNonlin Professional 6.3 software (Pharsight Corporation). From the concentration-time data, the maximum plasma concentration (*C*_max_), the time to maximum concentration (*T*_max_), the area under the plasma concentration-time curve from time zero to time of last measurable concentration (AUC_last_), the area under the plasma concentration-time curve from time zero to infinity (AUC_0-∞_), the elimination constant (*k*_el_), the half-life *t*_1/2_, and the mean residence time (MRT_last_) were estimated for each individual animal in each group.

To evaluate the total drug exposure in the fasted state, the area under the plasma concentration-time curve from time 0 to 12 h (AUC_0–12_) and the area under the plasma concentration-time curve from 12 h to 24 h (AUC_12–24_) were additionally calculated. To characterize the gastroretention effect of ER tablets, the time span that plasma concentration is equal to or greater than half the value of the maximum concentration (i.e., ≥50% *C*_max_) (*T*_50%_*C*_max_) or the half-value duration (HVD) was estimated (23). To evaluate the food effect on macozinone pharmacokinetic profile, the means of AUC_last_ and *C*_max_ of two macozinone ER tablets were compared according to the following ratios: AUC_last(fed)_/AUC_last(fasted)_ and *C*_max(fed)_/*C*_max(fasted)_.

The relative bioavailability (*F*_rel_) of the test dosage form of the extended-release tablet was calculated according to the following equation:

$$F_{\mathrm{rel}}=\frac{\mathrm{mean}\mathrm{AU}C_{\mathrm{last},\mathrm{ER}}}{\mathrm{mean}\mathrm{AU}C_{\mathrm{last},\mathrm{IR}}}\cdot \frac{\mathrm{Dos}e_{\mathrm{IR}}}{\mathrm{Dos}e_{\mathrm{ER}}}\cdot 100\text{\%}$$

### Statistical analysis.

Pharmacokinetic parameters are expressed as mean ± standard deviation (SD) and as median and range for five animals. The statistical significance in the differences of the means for AUC_0–∞_, AUC_last_, *C*_max_, MRT_last_, *T*_max_, *k*_el_, and *t*_1/2_ was determined using a one-way ANOVA with Tukey’s multiple-comparison test. To evaluate the food effect on macozinone bioavailability, AUC_0–∞_, AUC_last_, and *C*_max_ were log transformed and analyzed using an unpaired Student’s *t* test at a 95% confidence level. Results were considered significant when *P* < 0.05.

### Data availability.

The data supporting the findings of this study are available in the article and its supplemental materials.
