# Supplementary material for: Antituberculosis Macozinone Extended-Release Tablets To Enhance Bioavailability: a Pilot Pharmacokinetic Study in Beagle Dogs
Source: Microbiol Spectr. 2022 Dec 12;11(1):e02327-22. doi: 10.1128/spectrum.02327-22 (PMC9927148; doi:10.1128/spectrum.02327-22)
Supplement: Supplemental file 1 — Supplemental material. Download spectrum.02327-22-s0001.pdf, PDF file, 0.7 MB [file spectrum.02327-22-s0001.pdf]

# SUPPLEMENTARY MATERIALS

## Anti-Tuberculosis Macozinone Extended-Release Tablets to Enhance Bioavailability: a Pilot Pharmacokinetic Study in Beagle Dogs

Angela Koryakova[a], Victoria Shcherbakova[b*], Olga Riabova[c], Yurii Kazaishvili[b*], Roman Bolgarin[b*], Vadim Makarov[c#]

[a]Chemical Diversity Research Institute, Khimki, Russia

[b]Nearmedic Pharma LLC, Obninsk, Russia

[c]Federal Research Centre "Fundamentals of Biotechnology" of the Russian Academy of Sciences (Research Centre of Biotechnology RAS), Moscow, Russia

Running Head: Pharmacokinetics for Macozinone Extended-Release Tablets

#Address correspondence to Vadim Makarov, makarov@inbi.ras.ru

*Present address: Victoria Shcherbakova, Promomed Rus LLC, Moscow, Russia; Yurii Kazaishvili, Promomed Rus LLC, Moscow, Russia; Roman Bolgarin, R-Pharm LLC, Moscow, Russia

Angela Koryakova and Victoria Shcherbakova contributed equally to this work. Author order was determined alphabetically.

## FIGURES

**FIG S1**. Typical MRM chromatograms of macozinone (457/344 m/z, **A**, **B**) and IS (468/344 m/z, **C**, **D**) in standard dog plasma sample (macozinone concentration 0.5 ng/mL – LLOQ) (**A**, **C**) and blank dog plasma sample (**B**, **D**)

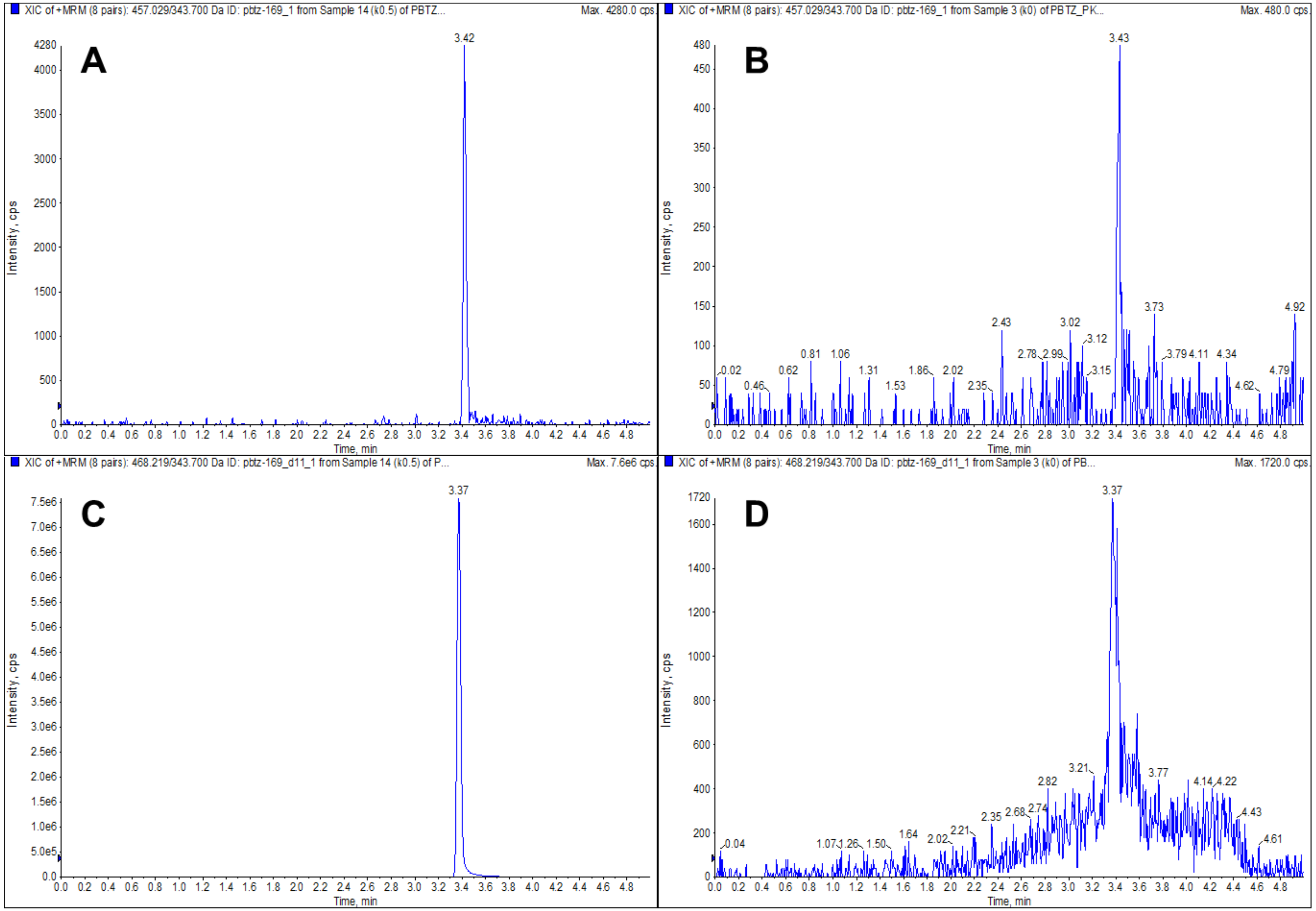

**FIG S2**. Individual plasma concentrations of macozinone extended-release tablets F2 (**A** – fasted state, **C** – fed state) and F6 (**B** – fasted state, **D** – fed state) after a single oral 500 mg dose in Beagle dogs (n = 5)

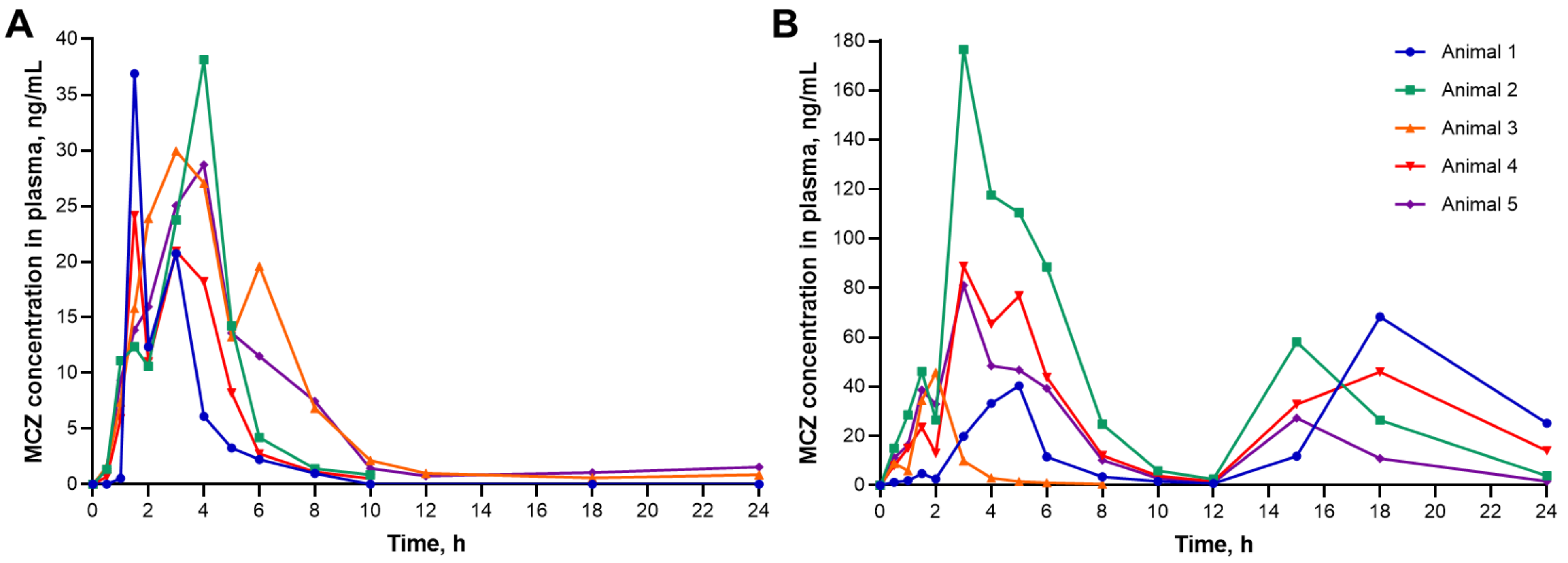

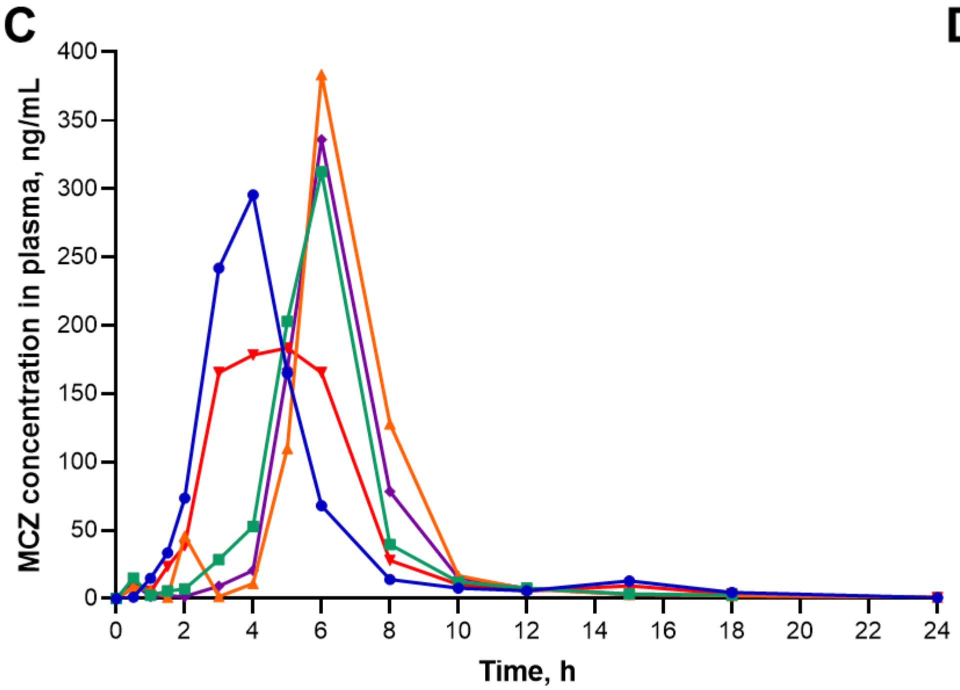
C
MCZ concentration in plasma, ng/mL
0
50
100
150
200
250
300
350
400
0 2 4 6 8 10 12 14 16 18 20 22 24
Time, h

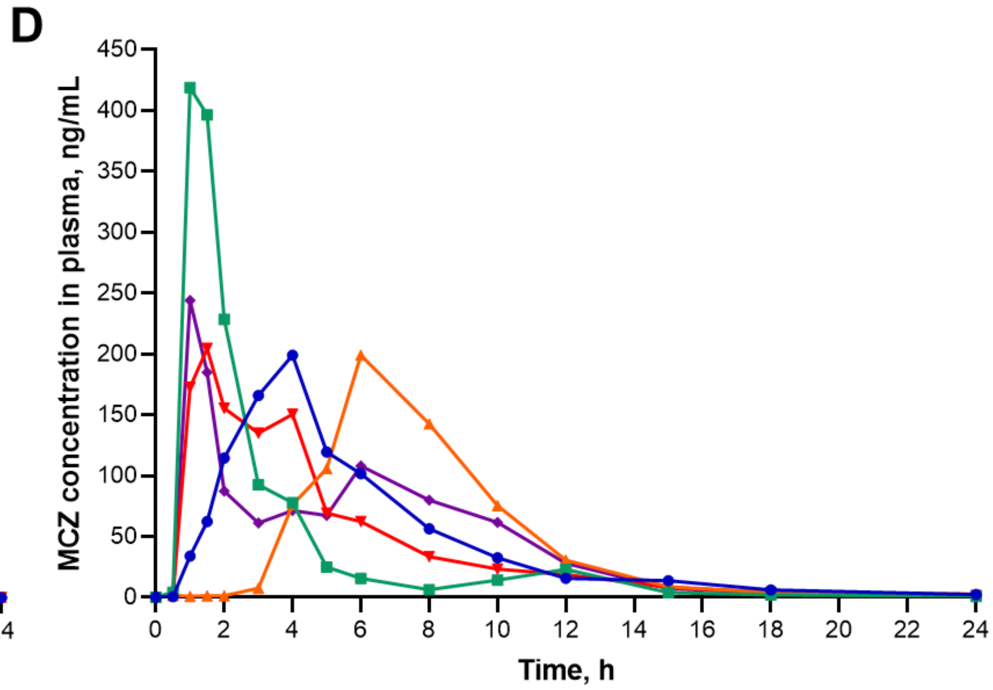
D
MCZ concentration in plasma, ng/mL
0
50
100
150
200
250
300
350
400
450
0 2 4 6 8 10 12 14 16 18 20 22 24
Time, h

**FIG S3**. Mean plasma concentrations of macozinone extended-release tablets F2 (**A**) and F6 (**B**) in fasted state (green line) vs. fed state (red line) after a single oral 500 mg dose in Beagle dogs (n = 5) (food effect)

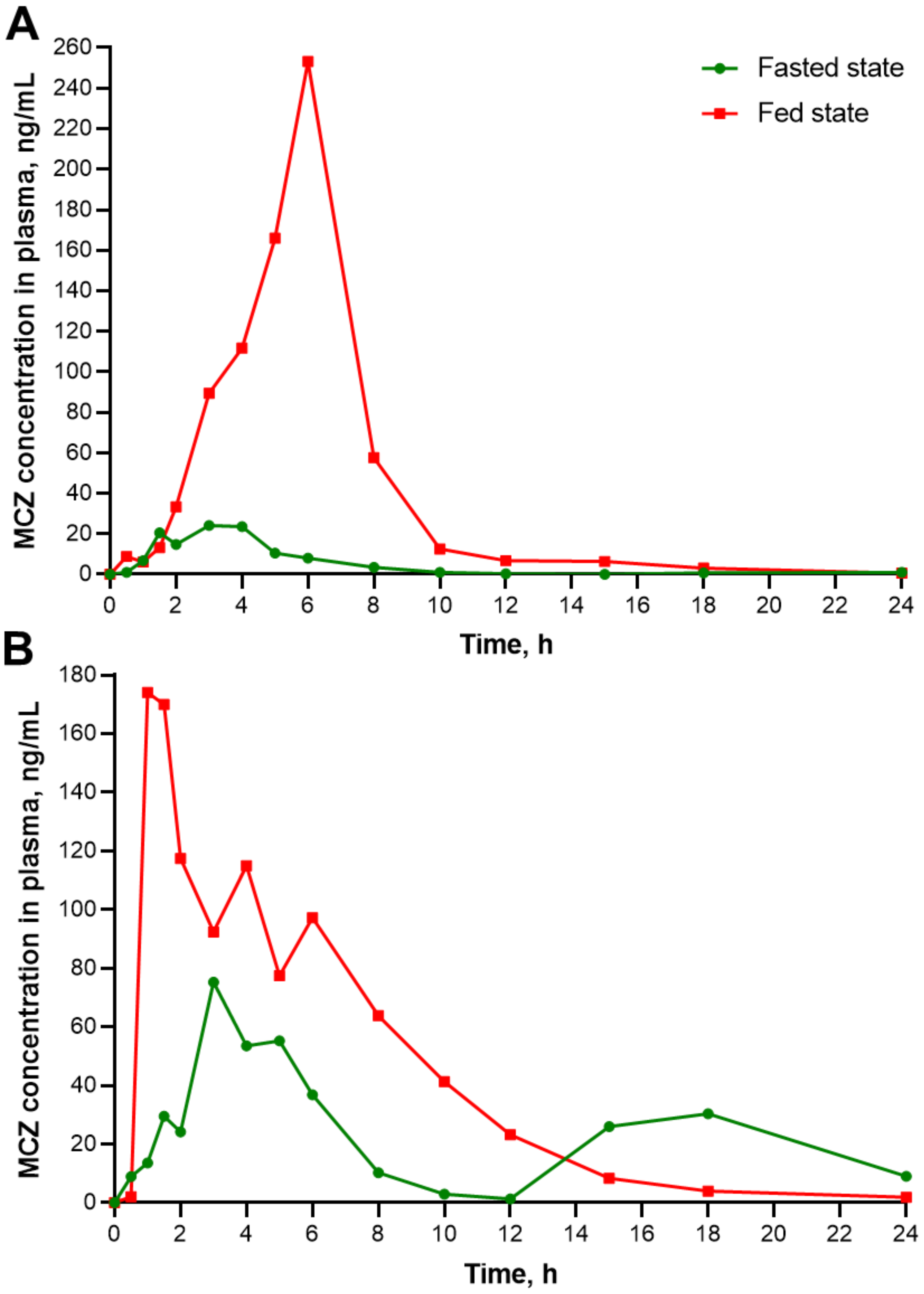

**TABLES**

**TABLE S1**. Summary of key *in vitro* characteristics of extended-release tablets F2 and F6 [1]

| **MCZ Oral formulation** | ***In vitro* parameter** | | | | | |
|---|---|---|---|---|---|---|
| | **Mean swelling index, %** | **Mean cumulative MCZ release in 0.1M HCl solution, %** | | | **Mean work of adhesion, $J/m^2$** | **Mean mucoadhesion time, h** |
| | | **1** | **3** | **6** | | |
| ER Tablet F2 | 105.1 | 2.1 | 5.5 | 26.1 | 7.0 | > 300 |
| ER Tablet F6 | 99.3 | 13.0 | 42.9 | 57.8 | 6.7 | > 300 |

**TABLE S2**. Areas under the plasma concentration-time curve from time zero to 12 h $AUC_{0-12}$ and from 12h to 24 h $AUC_{12-24}$ and their percentage of $AUC_{0-\infty}$ for extended-release tablets F2 and F6 in fasted state

| MCZ Oral formulation | Value | PK Parameter | | | |
|---|---|---|---|---|---|
| | | $AUC_{0-12}$ | | $AUC_{12-24}$ | |
| | | h·ng/mL | % of $AUC_{0-\infty}$ | h·ng/mL | % of $AUC_{0-\infty}$ |
| ER Tablet F2 | Mean±SD | 102.0±34.3 | 95.4±4.5 | | |
| | Median | 104.0 | 97.6 | - | - |
| | Range | 60.5-145.0 | 88.7-99.1 | | |
| ER Tablet F6 | Mean±SD | 301.0±227.0 | 59.5±32.8 | 228.0±162.0 | 36.0±28.4 |
| | Median | 311.0 | 59.3 | 282.0 | 38.4 |
| | Range | 70.9-353.0 | 20.7-98.8 | 126.0-399.0 | 28.7-67.3 |

## REFERENCES

1. Nesterenko, V.G.; Bolgarin, R.N.; Rudoy, B.A.; Salakhetdinov, D.K.; Kazaishvili, Yu.G.; Scherbakova, V.S.; Nikitina, N.A.; Medvedev, Yu.V.; Fisher, E.N.; Malashenko, E.A.; Shohin, I.E. Development of a gastro-retentive dosage form of a new promising anti-tuberculosis drug macozinone. *Razrabotka i registratsiya lekarstvennykh sredstv = Drug development & registration*, **2021**, *10*, 55-69.
